# Point-of-care ultrasound for monitoring catheter tip location during umbilical vein catheterization in neonates: a prospective study

**DOI:** 10.3389/fped.2023.1225087

**Published:** 2023-08-23

**Authors:** Hao-Qiang Xie, Cai-Xuan Xie, Jin-Feng Liao, Feng-Dan Xu, Bang Du, Bai-Mao Zhong, Xiao-Guang He, Ning Li

**Affiliations:** Department of Neonatology, Dongguan Children’s Hospital Affiliated to Guangdong Medical University, Dongguan, China

**Keywords:** point-of-care ultrasound (POCUS), umbilical vein catheterization, displacement, misalignment, neonates

## Abstract

**Background:**

Point-of-care ultrasound (POCUS) can guide umbilical vein catheter placement in real time and monitor catheter tip position, allowing avoidance of severe complications due to catheter malposition. This study aims to explore the effectiveness of POCUS in guiding venous catheter insertion and monitoring complications.

**Methods:**

Sixty-eight neonates with ultrasound-guided venous catheter insertion at the Neonatal Department of Dongguan Children's Hospital between December 2020 and February 2022 were included. POCUS was applied to monitor catheter tip location daily until catheter removal. A displacement range exceeding the intersection of the inferior vena cava and right atrium by ±0.5 cm was considered misalignment.

**Results:**

Sixty-four neonates had a displaced catheter tip (94.1%, 64/68), with a median displacement distance of 0.4 cm (minimum −0.2 cm, maximum 1.2 cm). Ten neonates had a misalignment (14.7%, 10/68) caused by displacement. Displacement usually occurs within 2–4 days after placement, with displacement rates of 94.1% (64/68), 90.6% (58/64), and 98.3% (59/60) on days 2, 3, and 4, respectively, and could still occur on day 9 post-placement. In addition, misalignment mainly occurs on the second day after placement. During the monitoring process, 58 neonates had catheter tip displacement ≥2 times, resulting in 252 displacement and 22 misalignment incidents. Among them, the catheter tip migrated outward from the inferior vena cava seven times, all of which were removed in time. Ultrasound was used for positioning 486 times, and x-ray was indirectly avoided 486 times.

**Conclusion:**

The catheter tip is prone to displacement and misalignment after umbilical vein catheterization, which most commonly occurs on days 2–4. POCUS is recommended for daily monitoring of the tip location during umbilical vein catheterization until catheter removal.

## 1. Introduction

**Figure 1:**
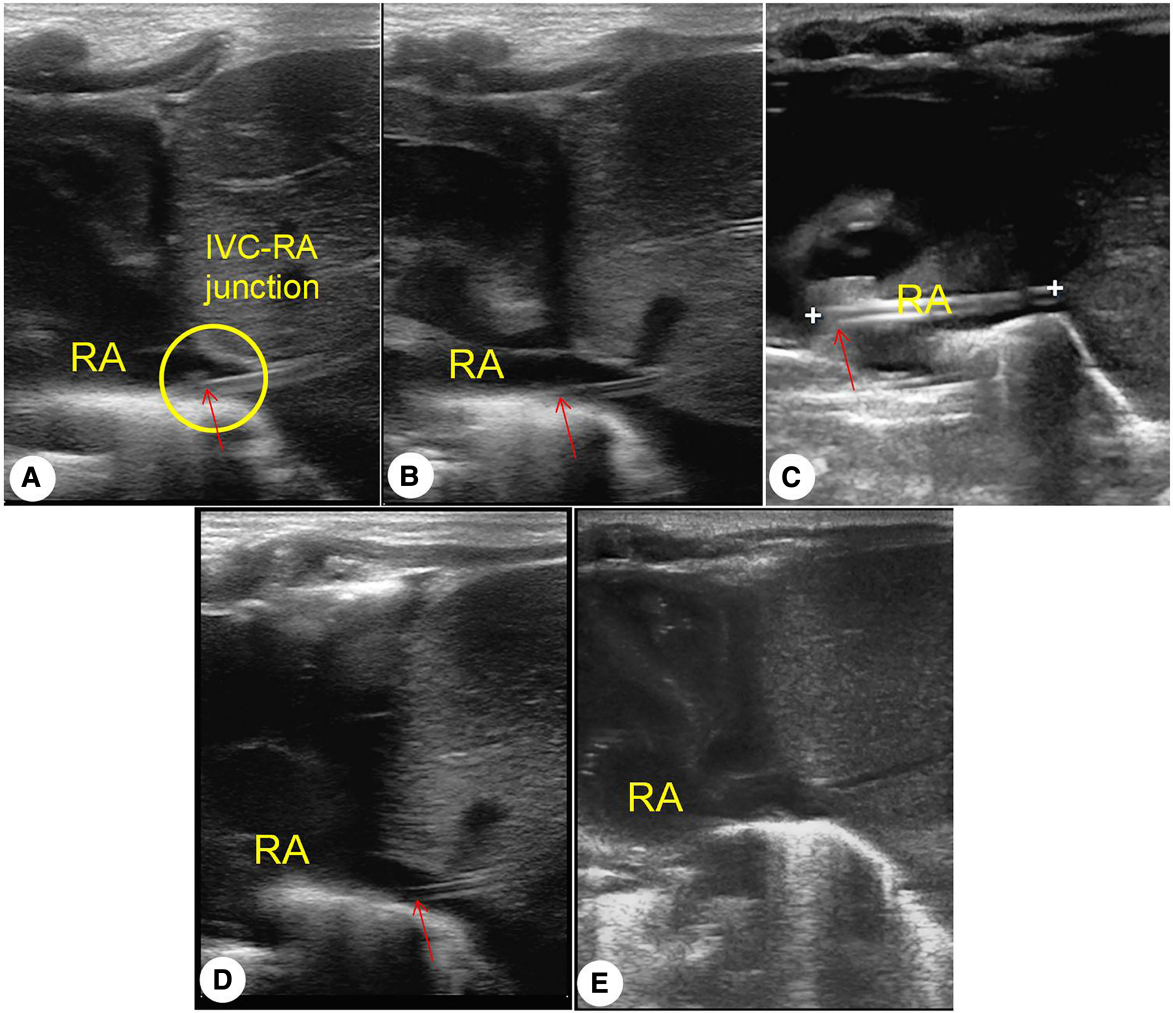
(**A**) standard positioning of umbilical vein catheterization: inferior vena cava–right atrium interchange. (**B**) Catheter tip is deeply displaced. (**C**) Catheter tip is deeply misaligned. (**D**) Catheter tip is shallowly displaced. (**E**) Catheter tip is shallowly misaligned. LA, left atrium; arrow, umbilical vein catheter tip.

Umbilical vein catheterization (UVC) is an important infusion channel in neonatal intensive care (1). Anatomical routes include the umbilical vein, extraperitoneal segment, intrahepatic segment, umbilical crypt, venous catheter, and inferior vena cava (IVC) (2). The ideal UVC tip location is outside the heart at the junction of the IVC and right atrium (RA) (3). However, the catheter tip is prone to post-catheterization displacement or even misalignment (4) (Figure 1). Chest radiography (CR) is the most commonly used tool to locate the catheter tip based on either the thoracic vertebral bodies or cardiac silhouette as landmarks, although a significant number of studies have questioned the accuracy of CR for this purpose (3).

Point-of-care ultrasound (POCUS) is a convenient, non-invasive modality that does not require radiation exposure. It can guide UVC in real time and monitor the catheter tip position (5). Early detection of catheter tip displacement can effectively prevent arrhythmia, pericardial effusion (6), pericardial tamponade (7), endocarditis, pleural effusion (8), liver hemorrhage, liver tissue necrosis (9), and other serious complications. In this study, 68 neonates with ultrasound-guided venous catheter insertion were prospectively assessed. POCUS was used to monitor the catheter tip position daily post-catheterization, and displacements, misalignments, and related catheter tip complications were recorded.

## 2. Patients and methods

### 2.1. Patients

This prospective study was approved by the Research Ethics Committee of Dongguan Children's Hospital (No. LL2022063002). The participants were 68 neonates who successfully underwent UVC under POCUS guidance in the Neonatology Department of Dongguan Children's Hospital between December 2020 and February 2022. The inclusion criteria for UVC (1) were the following: (1) small for gestational, weighing <1.5 kg, and needing long-term intravenous nutrition; (2) critically ill and requiring early fluid resuscitation and vasoactive drugs; and (3) underwent successful catheterization under ultrasonic guidance. Exclusion criteria were the following: (1) abnormal development of the umbilical cord and extremely low ligation position and (2) suffering from complications such as omphalitis, necrotizing enterocolitis (NEC), peritonitis, bleeding, and blood flow disorders in the lower limbs or buttocks. The umbilical stump needed to be kept at 1 cm during catheterization, which needed to have been completed within 24 h after birth.

### 2.2. Umbilical vein catheterization and location

**Figure 2:**
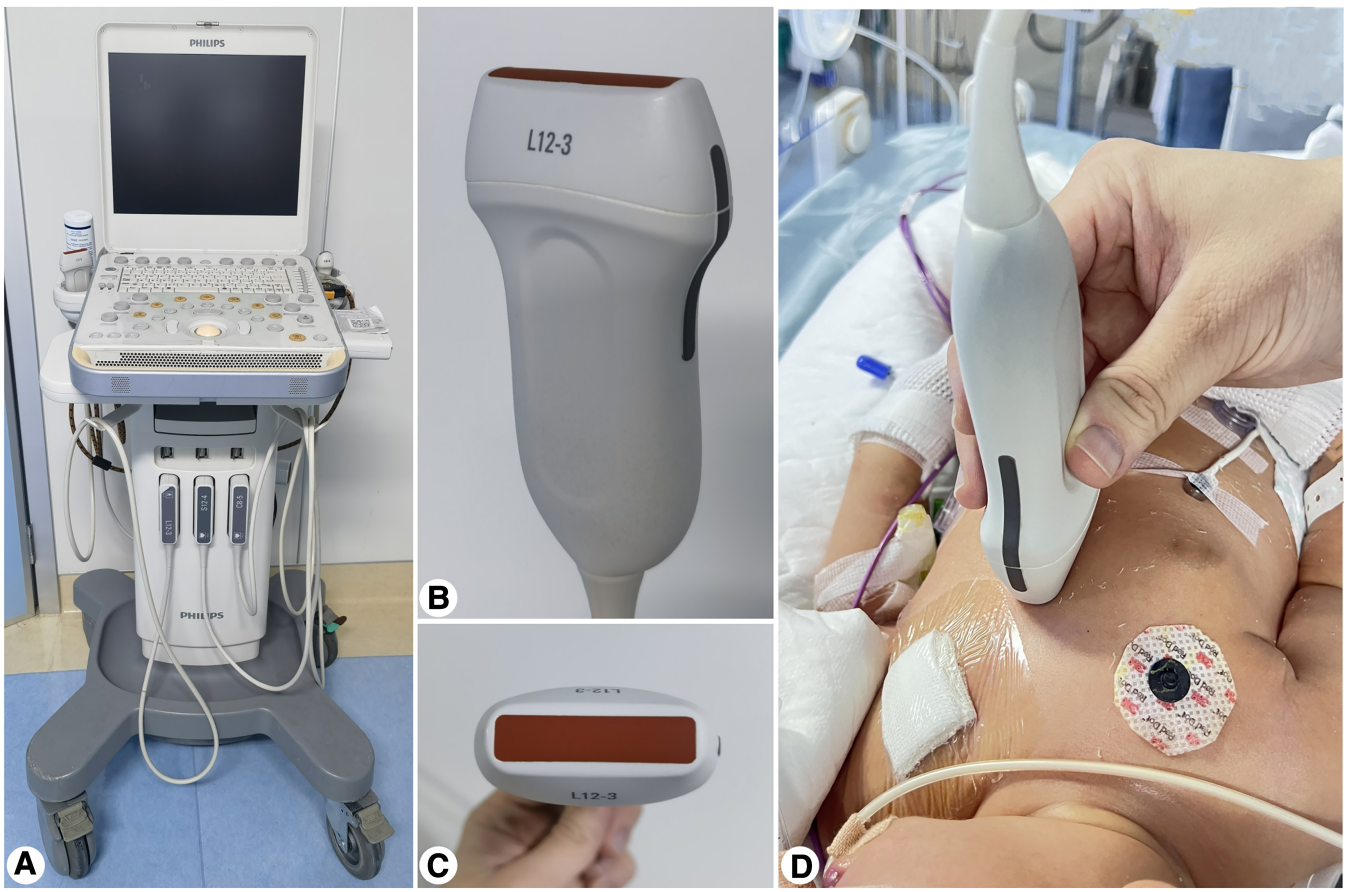
(**A**) Philips CX50 portable color ultrasound. (**B**,**C**) Linear array shallow small organ probe L 12–3 [probe type: broadband linear array probe; frequency range (MHz): 12–3]. (**D**) Bedside POCUS operation.

The insertion depth was calculated using the modified Shukla formula (weight formula) (10): preset insertion depth = [weight (kg) × 1.5+5.5]+umbilicalstumplength). A CX50 portable color ultrasound machine (Philips, CX50, USA), equipped with a linear array superficial small organ probe L12-3 (frequency range 12–3 MHz), was used to explore the catheter location (Figure 2). After UVC, the catheter tip was inserted into the IVC–RA intersection under POCUS guidance, injecting a small volume (0.5–2 ml) of saline to determine the needle tip location (3). The end of the external catheter was fixed when smooth blood flow was observed.

### 2.3. Observation indexes

#### 2.3.1. General clinical information

Patients’ general information, including sex, gestational age at birth, birthweight, major diagnoses, respiratory support mode at admission, UVC duration, complications, ultrasound-monitored distance between the catheter tip and standard positioning, directions and distances of catheter tip displacements, frequency of catheter tip displacements, misalignments, and displacement-caused misalignments, was collected.

#### 2.3.2. Displacement and misalignment of the umbilical vein catheter tip

After UVC, POCUS was used to monitor the catheter tip position every 24 h. Catheter tip position changes were considered displacements, and a displacement range exceeding ±0.5 cm at the IVC–RA intersection was regarded as a misalignment (11). Displacement directions and distances were recorded. The catheter tip was adjusted to the ideal position when there were no cardiac wall damage complications (e.g., pericardial effusion, pericardial tamponade) when ultrasound monitoring detected a catheter tip shift deeper than 0.5 cm or migration to the posterior RA wall.

#### 2.3.3. Statistical analysis

SPSS version 23.0 was used for data analysis. The normally distributed data are reported as $\bar{x}\pm s$, and non-normally distributed data are reported as median (minimum, maximum) and frequency (%).

## 3. Results

### 3.1. General clinical data

Among the 68 neonatal participants, 34 (50.0%) were male, with average gestational age of 33.4 ± 4.1 weeks and birthweight of 2,059.26 ± 842.43 g (Table 1). A total of 41 (60.3%) neonates had newborn respiratory distress syndrome (NRDS) (Table 2), and 53 (79.3%) neonates required mechanical ventilation (Table 3). All participants underwent UVC under ultrasound guidance within 24 h after birth, and the mean catheter use duration was 7.1 (6.0, 9.0) days. From December 2020 to February 2022, we used POCUS to guide umbilical vein catheterization and monitored the position of the catheter tip every day after catheterization until catheter extubation. Ultrasound was used 486 times for positioning. The number of UVC placements was perfectly monitored by POCUS, and no further x-ray examinations were acquired.

**Table 1:** General clinical data.

Factor	(*N* = 68)
Male, *n* (%)	34 (50.0)
Gestational age ($\bar{x}\pm s$, weeks)	33.4 ± 4.1
Birthweight ($\bar{x}\pm s$, g)	2,059.26 ± 842.43
Average duration of catheter use, M (P25, P75), days	7.1 (6.0, 9.0)

**Table 2:** Clinical disease factors.

Factor	Frequency	Percentage
Prematurity, *n* (%)	4	5.9
NRDS, *n* (%)	41	60.3
Asphyxia neonatorum, *n* (%)	6	8.8
MAS, *n* (%)	8	11.8
Pneumonia of newborn, *n* (%)	6	8.8
ARDS, *n* (%)	2	2.9
Congenital skin defect, *n* (%)	1	1.5
Total	68	100.0

MAS, neonatal aspiration of meconium; ARDS, acute respiratory distress syndrome.

**Table 3:** Respiratory support at admission.

Factor	Frequency	Percentage
Invasive mechanical ventilation	54	79.4
Non-invasive mechanical ventilation	11	16.2
Non-respiratory support	3	4.4
Total	67	100.0

### 3.2. Daily POCUS monitoring of catheter tip displacements and misalignments

Table 4 presents the daily post-catheterization POCUS monitoring data for the distance between the catheter tip and standard positioning and the catheter tip displacement distance. On the day of UVC, the catheter tip was in the standard, ultrasound-guided position in all cases, and no ultrasound-guided repositioning was repeated. On day 2 post-catheterization, catheter displacement was reported in 64 patients (94.1%, 64/68), among which 10 neonates (14.7%, 10/68) had dislocations caused by displacements. Displacements mostly occurred within 2–4 days post-placement, at incidence rates of 94.1% (64/68), 90.6% (58/64), and 98.3% (59/60) on days 2, 3, and 4, respectively. Misalignment most likely occurred on the second day after catheterization, at an incidence rate of 14.7% (10/68). The catheter tip was still misaligned on day 9 post-catheterization. During monitoring, 58 neonates had catheter tip displacement ≥2 times, resulting in 252 displacements and 22 misalignments. The catheter tip was dislocated deep to the posterior wall of the RA 13 times, and the catheter tip was dislocated shallow to the IVC seven times.

**Table 4:** Daily POCUS monitoring of catheter tip.

Factor	D1 (*n* = 68)	D2 (*n* = 68)	D3 (*n* = 64)	D4 (*n* = 60)	D5 (*n* = 58)	D6 (*n* = 52)	D7 (*n* = 42)	D8 (*n* = 29)	D9 (*n* = 22)	D10 (*n* = 13)	D11 (*n* = 8)	D12 (*n* = 2)	Total incidence (*n* = 486)
Distances between catheter tip and standard position ($\bar{x}\pm s$, cm)	0	0.37 ± 0.22	0.37 ± 0.16	0.24 ± 0.20	0.21 ± 0.21	0.17 ± 0.20	0.15 ± 0.21	0.21 ± 0.39	0.07 ± 0.28	0.17 ± 0.12	0.18 ± 0.12	0.20 ± 0.14	—
Catheter tip displacement distances ($\bar{x}\pm s$, cm)	0	0.37 ± 0.22	0.09 ± 0.19	−0.11 ± 0.14	−0.8 ± 0.1	−0.2 ± 0.1	0.0 ± 0.14	0.03 ± 0.2	−0.04 ± 0.14	−0.01 ± 0.03	0	0	—
Umbilical vein catheter tip displacement, *n*(%)	0	64 (94.1)	58 (90.6)	59 (98.3)	35 (60.3)	19 (36.5)	8 (19)	6 (21.4)	3 (13.6)	0	0	0	252 (51.9)
Umbilical vein catheter tip misalignment, *n* (%)	0	10 (14.7)	1 (1.6)	1 (1.7)	2 (3.4)	2 (3.8)	3 (7.1)	1 (3.6)	2 (9.1)	0	0	0	22 (4.53)

### 3.3. Physiologic changes with umbilical vein catheter tip misalignment

In 22 neonates with catheter tip misalignment, external catheter fixation was good, and the length of the exposed catheter did not change. When the catheter tip was misaligned in terms of depth, invasive mechanical ventilation was changed to non-invasive mechanical ventilation in seven patients (31.8%, 7/22). The average airway pressure decreased (2.5–4.6 cm H_2_O) in 10 patients (45.5%, 10/22), the umbilical stump dried to the root in four patients (18.2%, 4/22), and the abdominal circumference increased (more than 2 cm) in seven patients (31.8%, 7/22) when the catheter tip was misaligned.

### 3.4. Complications

The main complications of UVC included deep catheter tip displacement to the posterior RA wall, catheter tip displacement to the IVC, suspicion of NEC with increased abdominal distension, catheter-associated infection, pericardial tamponade, liver necrosis, and thrombosis. Deep catheter tip displacement to the posterior wall of the RA occurred in 13 patients on day 2 post-catheterization. In seven patients, the catheter tip shifted out of the IVC, including two (3.4%) on day 5 and two (9.1%) on day 9 post-catheterization. Suspicion of NEC with increased abdominal distension was observed in six patients. Catheter-associated infection occurred in one patient day 6 post-catheterization. Pericardial tamponade was reported in one patient on day 2 post-catheterization. No patient suffered from liver necrosis or thrombosis (Table 5).

**Table 5:** Complications from catheter tip with displacements and misalignments.

Factor	D1 (*n* = 68)	D2 (*n* = 68)	D3 (*n* = 64)	D4 (*n* = 60)	D5 (*n* = 58)	D6 (*n* = 52)	D7 (*n* = 42)	D8 (*n* = 28)	D9 (*n* = 22)	D10 (*n* = 13)	D11 (*n* = 8)	D12 (*n* = 2)	Total incidence
No incidence, *n* (%)	0	57 (83.8)	63 (98.4)	59 (98.3)	54 (93.1)	49 (94.2)	38 (90.5)	27 (96.4)	20 (90.9)	12 (92.3)	8 (100)	2 (100)	0
Catheter tip deeply displaced in posterior wall of right atrium, *n* (%)	0	9 (13.2)	1 (1.6)	0	0	1 (1.9)	2 (4.8)	0	0	0	0	0	13
Catheter tip shallowly displaced from the inferior vena cava, *n* (%)	0	0	0	1 (1.7)	2 (3.4)	0	1 (2.4)	1 (3.6)	2 (9.1)	0	0	0	7
Increased bloating, suspicion of NEC, *n* (%)	0	1 (1.5)	0	0	2 (3.4)	1 (1.9)	1 (2.4)	0	0	1 (7.7)	0	0	6
Catheter-associated infection, *n* (%)	0	0	0	0	0	1 (1.9)	0	0	0	0	0	0	1
Cardiac tamponade, *n* (%)	0	1 (1.5)	0	0	0	0	0	0	0	0	0	0	1
Hepatonecrosis, *n* (%)	0	0	0	0	0	0	0	0	0	0	0	0	0
Thrombogenesis, *n* (%)	0	0	0	0	0	0	0	0	0	0	0	0	0

## 4. Discussion

The data herein showed a catheter tip displacement rate as high as 94.1% on day 2 post-UVC. Frequent displacement likely leads to misalignment and related complications, among which deep misalignment of the catheter tip into the RA is common. Herein, this occurred in the posterior wall of the RA in 13 patients; heart rate and blood pressure decreases were difficult to correct in one of these patients due to sudden fluid resuscitation and ultrasound-confirmed pericardial tamponade, and the rest were with good external fixation and reported no obvious clinical discomfort, as confirmed by POCUS. POCUS, used for daily catheter tip misalignment monitoring, showed that displacement occurred within 9 days post-catheterization. This emphasizes the need for vigilance to catheter tip position changes, which should be assessed daily.

Before inserting the catheter, neonatologists will use a formula to calculate the expected length to be inserted, and Shukla's formula is currently commonly used in clinical practice. However, Shukla's formula has been shown to lead to higher rates of over-insertion of UVC; therefore, a revised formula has been suggested (12). Bedside x-ray imaging is required for positioning after conventional UVC catheterization (13) Guimar et al. (14). Some neonates need to be imaged several times for catheter tip position adjustments, increasing the risks of frequently moving critically ill neonates and exposing them to radiation. Thus, bedside x-ray is unconducive to continuous, real-time monitoring of catheter tip position. Hoellering et al. (14) reported that the sensitivity, specificity, and accuracy of monitoring catheter tip location by bedside x-ray are 45%, 87%, and 66%, respectively, indicating low efficacy of this method, which cannot solely be used to accurately and safely determine catheter tip location. Guimarães et al. (14) reported that bedside x-ray-based positioning was in the best location based on ultrasonic evaluation in only 27.16% of neonates. Hence, there is a significant safety risk to monitoring catheter tip position with bedside x-ray alone. In contrast, POCUS is convenient, non-invasive, radiation-free, and can guide UVC in real time without x-ray-based complications (16, 17). Saline agitation can help localize the tip, so recent literature suggests that ultrasound with saline contrast injection should be the gold standard for catheter tip localization (18). Daily monitoring of POCUS-guided catheterization and catheter tip position further supports its feasibility.

After umbilical vein catheterization, the position of the catheter tip is prone to change. Franta et al. (4) reported that nearly 50% of neonates with UVC had displacement within the first week and that only 38% of catheter tips positioned with x-ray were in the standard position based on bedside ultrasound during UVC. Catheter tip displacement exceeding ±0.5 cm at the IVC–RA intersection can cause misalignment. Dubbink-Verheij et al. (11) reported that catheter tip displacement identified by ultrasound monitoring occurred in 63% of neonates on the first day, midweek, and weekend after UVC. Herein, POCUS-guided UVC positioned the catheter tip at the IVC–RA intersection. Post-catheterization, POCUS was also used to monitor the catheter tip position daily. On day 2 post-catheterization, there were 64 cases of catheter tip displacement (94.1%, 64/68), and 10 cases of misalignment (14.7%, 10/68). The displacement rates were 94.1% (64/68), 90.6% (58/64), and 98.3% (59/60) on the days 2, 3, and 4 post-catheterization, respectively. Catheter tip displacement was still found on day 9 post-catheterization. During monitoring, catheter tip displacement occurred ≥2 times in 58 neonates, resulting in 252 catheter tip displacements and 22 catheter tip misalignments.

A misaligned UVC catheter tip can cause complications, including catheter tip misaligning deep to the posterior wall of the RA, shallow catheter tip misalignment of the IVC, pericardial tamponade, liver necrosis, and thrombosis (6–9). In this study, we found that catheter tip misalignment deep to the posterior wall of the RA occurred 13 times, mainly on the second day after catheterization. Among these, only one case of severe pericardial tamponade occurred. Misaligned withdrawal of the IVC from a shallow catheter tip occurred seven times, mainly during days 5–9 post-catheterization.

UVC catheter tip displacement can be affected by factors including pulmonary disease, diaphragm movement, mechanical ventilation pattern (19, 20), abdominal circumference change (21), and umbilical stump contracture (2). The main diseases among our sample were NRDS in 41 patients (60.3%, 41/68) and mechanical ventilation in 53 patients (79.3%, 53/68). External catheter fixation was good when the UVC catheter tip was misaligned and the length of the exposed catheter was unchanged. Seven patients (31.8%, 7/22) required a change from invasive to non-invasive mechanical ventilation. Decreased average airway pressure (2.5–4.6 cm H_2_O) occurred in 10 patients (45.5%, 10/22), and in 4 patients (18.2%, 4/22), the umbilical stump dried to the root. Increased abdominal circumference (>2 cm) occurred in seven patients (31.8%, 7/22). These cumulative data suggest that POCUS should be used to monitor catheter tip position daily and to make appropriate adjustments when there is improvement in pulmonary disease and abdominal conditions and when respiratory support changes.

The main funding was that POCUS should be mastered by standardized training according to relevant requirements and standards. Umbilical vein catheter tip positioning should also be closely monitored, and relevant parameters should be objectively recorded. However, note that the sample size of this study was limited; therefore, further data collection and analysis will be needed.

## 5. Conclusion

UVC catheter tip displacement and misalignment occurred most commonly on days 2–4 post-catheterization, potentially affected by factors including pulmonary disease, mechanical ventilation mode changes, changes in average airway pressure, increases or decreases in abdominal circumference, and dry contracture of the umbilical stump. Displacement was prone to misalignment and related serious complications. Catheter tip misalignment to the posterior wall of the RA was common, with significant safety risks. POCUS is relatively simple to operate, non-invasive, has no radiation exposure, and can monitor for changes in catheter tip position in real time to avoid serious complications. It should thus be used daily after catheterization until removal.

## Data Availability

The raw data supporting the conclusions of this article will be made available by the authors, without undue reservation.
